# Oral Cavity Lesions in Patients after Chemotherapy

**Published:** 2011-05-01

**Authors:** S Tirgar Tabari, S Sedaghat, H Naderi

**Affiliations:** 1 Department of Dermatology, Yahya-nejad Hospital, Babol University of Medical Sciences, Babol, Iran

**Keywords:** Oral cavity, Lesion, Chemotherapy

Dear Editor,

Oral lesions may be as a manifestation of mucocutaneous or systemic disease or a side effect of treatment [1] including laceration, erosion, vesicle, traumatic lesions (such as cheek biting, denture induced lesions), infections (candidiasis, warts,..), chemical or heat burn lesions, drug complications, radiotherapy effects and specific lesions in pemphigus vulgaris, lichen planus and aphtus stomatitis.[1][2][3][4][5]

Drug-induced lesions are very different and compromise xerostomy, stomatitis, erosions, bullous lesions similar to pemphigus, candidial or bacterial infections.[5][6][7] Oral lesions after chemotherapy were noticed in12-80% of patients. Good hygiene of oral cavity and teeth has important preventive role for these lesions. Oral mucositis is seen in 1/3 of patients with solid tumors. Leucopenia, fever and long duration chemotherapy are risk factors in induction of oral mucositis. Antifungal treatment with incomplete or complete gastrointestinal absorption can prevent oral candidiasis and reduce clinical features of the infection.[4][5][6][7] Oral candidiasis is one of the causes of morbidity in patients with acute leukemia after chemotherapy. It also increases the risk of esophagus candidiasis.[7]

Oral lesions after chemotherapy such as mucositis, oral bleeding, infections and xerostomy may be very severe and lead to interruption of chemotherapy. Mucositis is one important factor in limitation of upper limit of drug doses and commonest cause of morbidity in post-chemotherapy patients. [3] Dental decay, previous history of oral lesions, smoking, bad oral hygiene and some drug consumptions were shown as risk factors of mucositis. Antifungal drugs and ice chips have significant effects in prevention of mucositis after chemotherapy.[5][6]

This descriptive-analytical study was performed on 130 patients who underwent chemotherapy in Babol University of Medical Science, Northern Iran. A questionnaire was provided including information about sex, age and type of lesions before and after chemotherapy completed for every patient. Data were analyzed by SPSS software (version 11, Chicago, IL, USA) using t and Chi-Square tests. Sixty six (50.8%) did not have any lesions at admission time or after chemotherapy. Thirty three (25.4%) cases at the admission time and after chemotherapy had oral lesions and 31 (23.8%) cases after chemotherapy experienced oral lesions. Types of oral lesions before and after chemotherapy are depicted in Table 1.

One of the important points in our study was the significant correlation between age and oral complications before and after chemotherapy. Mean age of patients without oral lesions before and after chemotherapy was 48.7±17.6 years and mean age of cases that had oral lesions after chemotherapy was 58.8±11.7 years (p=0.005). Old age was one of the important risk factor for oral lesions after chemotherapy. Type of cancer did not have a significant correlation with oral lesions. Before chemotherapy, 22.8% of cases of hematological cancers, 22.9% of gastrointestinal carcinoma and 45.5% patients with other carcinomas had oral lesions. After chemotherapy, 50% of patients with hematological cancers, 44.4% with gastrointestinal carcinoma and 54.5% with other cancers had oral lesions (p=0.82).

Oral lesions are one of the most common complications of chemotherapy. So every chemotherapy center needs trained medical staffs for diagnosis, prevention and control of oral cavity lesions and careful attention to age of patients under chemotherapeutic courses could prevent severe oral complications.

**Table 1:** Prevalence and percent of oral lesions in patients undergoing chemotherapy in Babol Medical Science University, Iran.

****	**Number of patients**		****
**Type of lesions**	**Before chemotherapy No (%)**	**After chemotherapy No (%)**
Ulcer	4 (3.1)	7 (5.4)
Perlech	5 (3.8)	17 (13.1)
Trush	4 (3.1)	34 (26.2)
Leucoplakia	0 (0)	3 (2.3)
Loss of lingual papilla	15 (11.5)	16 (12.3)
Inflammation of oral mucosa	22 (16.9)	39 (39.0)
